# Integrative approach identifies *SLC6A20* and *CXCR6* as putative causal genes for the COVID-19 GWAS signal in the 3p21.31 locus

**DOI:** 10.1186/s13059-021-02454-4

**Published:** 2021-08-23

**Authors:** Silva Kasela, Zharko Daniloski, Sailalitha Bollepalli, Tristan X. Jordan, Benjamin R. tenOever, Neville E. Sanjana, Tuuli Lappalainen

**Affiliations:** 1 New York Genome Center, New York, NY USA; 2 Department of Systems Biology, Columbia University, New York, NY USA; 3 Department of Biology, New York University, New York, NY USA; 4 Department of Microbiology, Icahn School of Medicine at Mount Sinai, New York, NY USA

**Keywords:** COVID-19, SARS-CoV-2, GWAS, eQTL, CRISPR

## Abstract

To date, the locus with the most robust human genetic association to COVID-19 severity is 3p21.31. Here, we integrate genome-scale CRISPR loss-of-function screens and eQTLs in diverse cell types and tissues to pinpoint genes underlying COVID-19 risk. Our findings identify *SLC6A20* and *CXCR6* as putative causal genes that modulate COVID-19 risk and highlight the usefulness of this integrative approach to bridge the divide between *correlational* and *causal* studies of human biology.

**Supplementary Information:**

The online version contains supplementary material available at 10.1186/s13059-021-02454-4.

## Background

COVID-19, which is caused by SARS-CoV-2 (severe acute respiratory syndrome coronavirus 2) infection, results in diverse individual disease courses from asymptotic carriers to severe disease with respiratory failure [1, 2]. A growing body of evidence suggests also an important role of genetic factors in COVID-19 susceptibility and severity [3–5]. Most notably, the chromosome 3p21.31 locus was one of the first genome-wide significant loci found to be associated with COVID-19 [3]. To date, 3p21.31 encompasses the strongest and the most replicated signal for more severe outcomes of SARS-CoV-2 infection. However, the functional mechanisms of this association are unclear. The locus includes multiple protein-coding genes, for example, *LIMD1*, *SACM1L*, *SLC6A20*, *LZTFL1*, *CCR9*, *FYCO1*, *CXCR6*, and *XCR1*, many of which have a potentially relevant role in the pathophysiology of COVID-19. As genetic variants have been shown to often exert their effect on complex traits or disease via *cis*-regulation of gene expression [6, 7], expression quantitative trait locus (eQTL) mapping could serve as a means to pinpoint candidate genes for traits or diseases of interest.

We previously performed a genome-scale CRISPR loss-of-function screen to identify genes required for SARS-CoV-2 viral infection in human lung epithelial-like cells expressing *ACE2* (A549^ACE2^) [8]. Top-ranked genes from this CRISPR screen have an established link in SARS-CoV-2 infection in a cell line and could be informative for prioritizing host genes for COVID-19. In this work, we present an integrative approach using the results of the CRISPR screen and eQTLs in various cell types and tissues from the eQTL Catalogue [9] and Genotype Tissue Expression (GTEx) v8 [7] data release to pinpoint genes underlying COVID-19 risk in the 3p21.31 locus. Our results suggest that genes enriched in the in vitro CRISPR screen contribute to COVID-19 risk in humans, and highlight *SLC6A20* and *CXCR6* as the putative causal genes in the 3p21.31 COVID-19 risk locus.

## Results and discussion

**Fig. 1:**
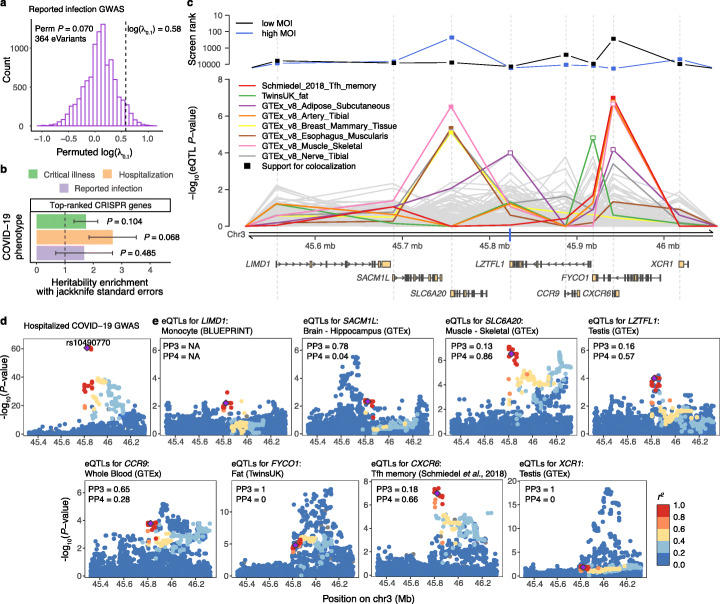
Genetic regulatory effects of top-ranked CRISPR genes and prioritization of genes in the 3p21.31 locus associated with COVID-19 GWAS. **a** Histogram of the permuted log(*λ*_0.1_) to test the significance of the inflation in reported SARS-CoV-2 infection GWAS for variants that are *cis-*eQTLs for top-ranked CRISPR genes in Lung (*n* = 364). Vertical dashed line denotes the observed log(*λ*_0.1_) value. **b** Heritability enrichment in top-ranked genes from the CRISPR screen for the three main COVID-19 phenotypes. Vertical dashed line denotes no enrichment. **c** Prioritization of genes in the 3p21.31 locus by integrating CRISPR screens and *cis*-eQTLs. The top panel shows the ranking of the genes in the locus according to the second-best guide RNA score in the low MOI (black) and high MOI (blue) pooled CRISPR screens. The middle panel shows the *cis*-eQTL *P* values for the lead GWAS variant in the 3p21.31 locus (rs10490770, denoted as a blue tick on the *x*-axis) in different cell types and tissues from the eQTL Catalogue and GTEx, 112 eQTL data sets in total. Highlighted are eight cell types/tissues, where the *cis*-eQTL *P* value for the lead GWAS variant is < 10^−4^ for at least one gene in the region. Filled square denotes support for colocalization between the GWAS and *cis*-eQTL signal (posterior probability for one shared causal variant (PP4) > 0.5). The bottom panel depicts the transcripts of the eight protein-coding genes in the locus. Ranks and *cis*-eQTL *P* values are aligned to match the start of the gene which is shown as a gray dashed line across the panels. **d**, **e** Regional association plots of the hospitalized COVID-19 GWAS (**d**) and *cis*-eQTLs for the eight genes (**e**) from the associated locus in the cell type/tissue where the lead GWAS variant has the lowest *cis*-eQTL *P* value. Purple diamond denotes the lead GWAS variant, and the data points are colored based on the (weighted average) LD between the lead GWAS variant and other variants in the region in the respective study population. PP3 and PP4—posterior probability for two different variants or one shared causal variant in coloc, respectively

To set the stage for identifying the causal genes for COVID-19 in the 3p21.31 locus, we first evaluated whether the top-ranked genes from the CRISPR screen in human lung cells contribute to COVID-19 risk in humans. We hypothesized that genetic regulatory variants for genes pinpointed by the CRISPR screen would show an increased signal for the genetic association for COVID-19 in genome-wide association studies (GWAS) in the human population. To this end, we analyzed if *cis*-eQTLs from lung tissue [7] for these genes are enriched for overall association signal in data from the COVID-19 Host Genetics Initiative (HGI) [5] for the three main COVID-19-related phenotypes: critical illness (A2), hospitalization (B2), reported infection (C2) as compared to population controls without known SARS-CoV-2 infection. Indeed, we observed a trend towards significance in inflation of the GWAS association signal for variants that are *cis*-eQTLs for the top-ranked genes from the CRISPR screen in the SARS-CoV-2 reported infection GWAS, compared to all lung *cis*-eQTLs (*λ*_0.1_ = 1.78, permutation *P* value = 0.070, Fig. 1a). We did not see a significant signal for the other two COVID-19 phenotypes in the present data (Additional file 1: Figure S1). Additionally, by using stratified linkage disequilibrium (LD) score regression [10], we noticed a suggestive signal for enriched heritability in the top-ranked genes for hospitalization conditional on the 96 annotations in the baseline model and the set of all protein-coding genes (*P* = 0.068, Fig. 1b). Thus, our results suggest the link between genes enriched in the *in vitro* CRISPR screen and the genetic component of COVID-19 risk in humans, but future COVID-19 GWAS with larger sample size are needed to refine this suggested association.

To elucidate which genes in the chromosome 3 locus might mediate the genetic association, we first observed that the Solute Carrier Family 6 Member 20 (*SLC6A20*) gene and the C-X-C Motif Chemokine Receptor 6 (*CXCR6*) gene have a relatively high rank in our CRISPR screen (Fig. 1c). Next, we analyzed if the COVID-19 hospitalization GWAS lead variant rs10490770, where the alternative allele increases risk, affects expression of any of these genes in the locus. Using data from the eQTL Catalogue [9] and GTEx v8 [7], we analyzed all *cis*-eQTL associations for this variant and performed colocalization analysis [11, 12] to assess if the *cis*-eQTL and GWAS signal share a genetic cause. *CXCR6* and *SLC6A20* stood out, with the eQTL data indicating that the COVID-19 severity variant affects the expression of these two genes (Fig. 1c, Additional file 1: Figure S2). The *cis*-eQTL associated with lower expression of *CXCR6* is active in memory T follicular helper (Tfh) CD4^+^ T cells [13] and colocalizes with the GWAS (PP4 = 0.66, Fig. 1d–e). The colocalization signal was very strong for the *cis*-eQTL associated with higher expression of *SLC6A20* in four tissues from GTEx - breast mammary tissue, esophagus muscularis, skeletal muscle, and tibial nerve (0.70 < PP4 < 0.90, Additional file 1: Figure S3, Additional file 2: Table S1). Other genes in the region, such as *FYCO1* and *XCR1*, also have regulatory variants affecting their expression, but two distinct variants likely drive the GWAS and eQTL signals (Fig. 1d–e). Also, we applied joint likelihood mapping (JLIM) [14] and corroborated the evidence for shared genetic effects of *cis*-eQTLs for *CXCR6* and *SLC6A20* and COVID-19 hospitalization (Additional file 1: Figure S4, Additional file 2: Table S2). Of note, colocalization of *cis*-eQTLs for *LZTFL1* in the testis and hospitalization GWAS with coloc was not replicated using JLIM (*P*_JLIM_ > 0.05/8). Additionally, we did not observe evidence for colocalization signal with splicing QTLs (sQTLs) nor transcript usage QTLs (tuQTLs) in *cis* (Additional file 2: Table S3). To confirm the robustness of the observed colocalization signal, we showed that the COVID-19 hospitalization signal was observed in the reported infection GWAS (Additional file 1: Figure S5). Given that both the CRISPR screen and eQTL data support the causal role of *SLC6A20* and *CXCR6*, it is possible that the COVID-19 GWAS association in the 3p21.31 locus is driven by pleiotropic effects of the same variant on multiple genes in different cell types.

In addition to human genetic evidence and support from experimental data, both *SLC6A20* and *CXCR6* have a plausible biological function that could affect COVID-19. Notably, SLC6A20 functionally interacts with ACE2 [15], the receptor of the SARS-CoV-2 Spike protein that is the key host gene for viral entry [16]. In contrast, CXCR6 regulates the localization of resident memory T (T_RM_) cells in the lung and maintains a pool of airway T_RM_ cells, critical for cellular immunity against respiratory pathogens [17]. It is also an alternate coreceptor for HIV [18, 19], raising a hypothesis of a similar function in lung cells for SARS-CoV-2. The fact that we observed *cis*-eQTLs for *SLC6A20* and *CXCR6* in different tissues and cell types other than lung highlights the complexity and possibility of extrapulmonary spread of SARS-CoV-2 [20], as well as potential pleiotropic effects of these genes in multiple cell types and physiological processes leading to COVID-19. However, further research is needed to establish whether all these *cis*-eQTLs have a causal role in COVID-19 risk. Importantly, GWAS can only show associations for common variants in the human population, and thus, the functional role of these genes in lung cells, captured by the screen, may not be captured by GWAS and *cis*-eQTL data.

Notably, in addition to the well-replicated signal for COVID-19 severity in the 3p21.31 locus, other independent signals more strongly associated with susceptibility to SARS-CoV-2 infection in the same region have been identified as sample sizes for COVID-19 GWAS have grown [21]. The two candidate genes based on our integrative analysis, *SLC6A20* and *CXCR6*, have been prioritized for reported infection and more severe outcomes of SARS-CoV-2 infection, respectively [5]. Corroborating evidence from entirely independent approaches pinpointing *SLC6A20* and *CXCR6* presented here, together with additional evidence from other studies, warrants further research into exploring the virological or mechanistic mode of action of these two genes in understanding their role in COVID-19 susceptibility and severity.

## Conclusions

In this work, our genome-wide analysis suggested that genes required for SARS-CoV-2 infection *in vitro* also contribute to COVID-19 susceptibility in humans. By integrating the results of CRISPR screen and *cis*-eQTLs, we have identified *SLC6A20* and *CXCR6* as potential protein-coding genes in the 3p21.31 locus through which noncoding variants associated with COVID-19 risk in human patients may function. This integrative approach should prove useful for other human diseases and pathogens to bridge the divide between *correlational* and *causal* studies of human biology.

## Methods

### Genome-wide CRISPR screen and guide RNA analysis

Details regarding the SARS-CoV-2 CRISPR screen in A549 human lung epithelial cells that over-express ACE2 (A549^ACE2^) have been described before [8]. Briefly, human GeCKOv2 A and B libraries (Addgene, 1000000048) were used for the genome-wide CRISPR-Cas9 screen [22]. After transduction and selection for the GeCKOv2 library (maintaining ~ 1000-fold library representation), we infected 125 million A549^ACE2^ cells with SARS-CoV-2 virus (NIAID BEI isolate USA-WA1/2020) at two multiplicity of infections (MOIs): 0.01 (low MOI) and 0.3 (high MOI), which differ by the amount of virus used to infect the cells. Surviving cells were collected on day 6 post-infection, when genomic DNA was isolated, guide RNAs were recovered by PCR, and guide representation was determined by next-generation sequencing (Illumina).

We aligned trimmed sequencing reads to the GeCKOv2 reference using bowtie v1.1.2 [-a --best --strata -v 1 –norc] allowing 1 nucleotide mismatch to determine the read counts per guide. Alignment rates were ~ 80% for all samples. We normalized read counts between biological samples and computed a fold-change by comparison of SARS-CoV-2-infected samples to the uninfected control. To identify genes where loss-of-function mutations reduce SARS-CoV-2 infection resulting in enrichment within the pool, the genes were ranked based on three methods: robust-rank aggregation (RRA) [23], RIGER (weighted-sum), and second-best rank method. For example, according to the second-best rank method, each gene in the human GeCKOv2 A+B library is targeted by 6 different guides. Genes are ranked based on the guide RNA with second highest fold-change (sbScore), as described before [24–26].

For enrichment analyses, we used a set of top-ranked (top500) genes in low MOI condition by each of three ranking methods (RRA, RIGER, sbScore), see Figure S1C in Daniloski et al. [8], resulting in *n* = 890 unique protein-coding genes across the methods.

### COVID-19 phenotypes and GWAS

We used summary statistics of the three main COVID-19 GWAS generated by the COVID-19 HGI [27] based on round 5, worldwide meta-analyses without 23andMe, released January 18, 2021 [5]: (1) A2 (critical illness)—very severe respiratory confirmed COVID vs. population (*n*_cases_ = 5582, *n*_controls_ = 709,010), (2) B2 (hospitalization)—hospitalized covid vs. population (*n*_cases_ = 12,888, *n*_controls_ = 1,295,966), (3) C2 (reported infection)—COVID vs. population (*n*_cases_ = 36,590, *n*_controls_ = 1,668,938). As the lead GWAS variant in the 3p21.31 locus to highlight on figures, we used rs10490770 (3:45823240:T:C), which had the lowest *P* value in the hospitalization COVID-19 worldwide meta-analysis with 23andMe as reported by the COVID-19 HGI [5].

### Inflation of COVID-19 GWAS signal for variants that are *cis*-eQTLs

To estimate the importance of the genes enriched in the CRISPR screen in modulating the COVID-19 risk in humans, we tested if we observe inflation of signal in the aforementioned COVID-19 GWAS for variants that are *cis*-eQTLs in GTEx v8 lung [7] for the top-ranked genes from the CRISPR screen. We measured inflation using the lambda inflation statistic relative to the chi-square quantile function of 0.1, *λ*_0.1_, i.e., estimating the inflation among 10% of the most significant tests. We calculated *λ*_0.1_ for two sets of *cis*-eQTLs: (1) all lead *cis*-eQTLs in the lung (9557 protein-coding genes with significant *cis*-eQTLs in the lung at false discovery rate (FDR) < 0.05, serving as a background set), (2) all lead *cis*-eQTLs in the lung for the top-ranked genes from the CRISPR screen (439 out of 890 top-ranked protein-coding genes have *cis*-eQTLs in the lung with FDR < 0.05).

To test the significance of *λ*_0.1_, we used a permutation-based test. We selected *n* number of lead *cis*-eQTLs from the background set *k* = 10,000 times, where *n* is the number of lead *cis*-eQTLs for the top-ranked genes from the CRISPR screen tested in a given COVID-19 GWAS, and calculated *λ*_0.1_ on the permuted data. To calculate two-sided permutation *P* values, we applied log-transformation on the permuted *λ*_0.1_ values to get a symmetric null distribution. We then calculated permutation *P* value as the proportion of permuted log(*λ*_0.1_) as extreme as or more extreme than the observed log(*λ*_0.1_).

### Partitioned heritability analysis

We performed stratified LD score regression (LDSC) [10] to calculate the single nucleotide polymorphism (SNP) heritability of COVID-19 phenotypes in the top-ranked genes from the CRISPR screen using GWAS summary statistics from the European-only meta-analysis (round 5, excluding 23andMe): A2—critical illness (*n*_cases_ = 4606, *n*_controls_ = 702,801), B2—hospitalization (*n*_cases_ = 9373, *n*_controls_ = 1,197,256), and C2—reported infection (*n*_cases_ = 29,071, *n*_controls_ = 1,559,712). We generated genome-wide custom annotation files and LD scores using the top-ranked genes identified in the CRISPR screen (*n* = 890 protein-coding genes across the three ranking methods) and used all protein-coding genes as background (--gene-coord-file in make_annot.py from LDSC package). We added 100 kb windows on either side of the transcribed region of each gene. Next, we jointly modeled the annotation corresponding to the top-ranked genes and the 96 annotations in the “baseline model” (*baselineLD_v2.2.tgz*, https://alkesgroup.broadinstitute.org/LDSCORE/GRCh38/). We have used regression weights (*weights.tgz*, https://alkesgroup.broadinstitute.org/LDSCORE/GRCh38/) from HapMap3 SNPs, excluding the HLA region and restricted to SNPs with minor allele frequency (MAF) > 5% (*plink_files.tgz*, https://alkesgroup.broadinstitute.org/LDSCORE/GRCh38/).

### Prioritization of genes in the 3p21.31 locus based on CRISPR screen and eQTL data

We focused on eight genes, *LIMD1*, *SACM1L*, *SLC6A20*, *LZTFL1*, *CCR9*, *FYCO1*, *CXCR6*, and *XCR1*, in the 3p21.31 locus that has been shown to robustly associate with COVID-19 severity [3–5]. Firstly, we ranked the genes based on the sbScore in the low MOI and high MOI CRISPR screen. Secondly, we gathered summary statistics for 112 *cis-*eQTL data sets from the eQTL Catalogue [9] (63 data sets, mostly immune cell types with and without treatment) and GTEx v8 [7] (49 tissues). We ranked the genes based on the *P* value in eQTL studies for the lead GWAS variant in the 3p21.31 locus. Since observing low eQTL *P* value for the lead GWAS variant does not necessarily translate into shared genetic causality, we further performed colocalization analysis.

### Colocalization analysis of COVID-19 GWAS and *cis*-eQTLs in the 3p21.31 locus with coloc

To assess evidence for shared causal variant of a *cis*-eQTL and a COVID-19 GWAS (hospitalized and reported infection GWAS, round 5, worldwide meta-analysis without 23andMe), we used the Bayesian statistical test for colocalization, coloc [11], assuming one causal variant per trait. We only included *cis*-eQTLs for genes for colocalization test, if there was a *cis*-eQTL with a nominal *P* value < 10^−4^ within 100 kb of the lead GWAS variant in the 3p21.31 locus. Coloc was run on a 1 Mb region centered on the lead GWAS variant (+/− 500 kb from the variant) with prior probabilities set to *p*_1_ = 10^−4^, *p*_2_ = 10^−4^, *p*_3_ = 5 × 10^−6^. We defined suggestive support for colocalization between the COVID-19 GWAS and *cis*-eQTL signal if the posterior probability for one shared causal variant (PP4) > 0.5.

Allelic heterogeneity of gene expression in *cis* is widespread [7], and it violates the assumption of one causal variant per trait. Thus, we also used the development version of coloc [12] (https://github.com/chr1swallace/coloc/tree/condmask) in a wider 2 Mb region centered at the lead hospitalized COVID-19 GWAS variant. The enhanced version of coloc allows conditioning and masking to overcome one single causal variant assumption. For eQTL data sets from the eQTL Catalogue, we used method = mask with LD data from the 1000 Genomes Project [28] EUR population that matches the genetic ancestry of the study population in the majority of the studies in the eQTL Catalogue. For eQTL data sets from the GTEx Project, we used method = cond with LD calculated from the individuals that had gene expression data in the given tissue in GTEx. We used the mode = iterative and allowed for a maximum of three variants to condition/mask. We set the *r*^2^ threshold to 0.01 to call two signals independent when masking, and we set the *P* value threshold to 10^−4^ to call the secondary signal significant. We used method = single for the hospitalized COVID-19 GWAS. As a result, the maximum PP4 (posterior probability for shared causal variants) estimates with conditioning/masking were similar to PP4 estimates from the standard coloc run (Additional file 2: Table S1), suggesting no additional colocalization events with secondary independent *cis*-eQTLs.

Additionally, standard coloc with the options mentioned above was used to test for colocalization between COVID-19 hospitalization and *cis*-sQTLs from GTEx v8 and *cis*-tuQTLs from the eQTL Catalogue (including both transcript usage and txrevise event usage). Note that *cis*-tuQTLs were mapped also for the GTEx tissues by the eQTL Catalogue.

### Colocalization analysis of COVID-19 GWAS and *cis*-eQTLs in the 3p21.31 locus with JLIM

To corroborate the observed colocalization signal found with coloc, we used JLIM (version 2.5) [14] with default options (https://github.com/cotsapaslab/jlim/). We treated hospitalized COVID-19 GWAS (round 5, European-only meta-analysis without 23andMe) as the primary trait and *cis-*eQTLs from GTEx v8 (mapped in European-American subjects) and the eQTL Catalogue (note that some studies are not from the European population) as the secondary trait. Reference genotype panel was created by including all the European subpopulations from the 1000 Genomes Project. By default, the analysis window was set to 200 kb, centered at the lead GWAS variant in the 3p21.31 locus.

### Regional association plots for the 3p21.31 locus

Data points in the locuszoom-like regional association plots are colored by the LD between the lead GWAS variant in the 3p21.31 locus (rs10490770) and other variants in the region. For plotting data for COVID-19 GWAS and *cis-*eQTLs from the eQTL Catalogue, we used genotype data from the 1000 Genomes Project, and calculated weighted average *r*^2^ value based on the counts of global ancestral populations in the analysis (if multiple global ancestry populations were studied). Note that for European populations, we only included non-Finnish populations in the 1000 Genomes Project. For plotting data for *cis-*eQTLs from the GTEx project, we used genotype data available via dbGaP, accession phs000424.v8, and calculated *r*^2^ using the set of individuals that had expression data from the given tissue.

## Supplementary Information

**Additional file 1:.** Supplementary figures S1-S5.

**Additional file 2:.** Supplementary tables S1-S3.

**Additional file 3.** Peer review history.

## Data Availability

The data sets analyzed during the current study are available from the following repositories: CRISPR screen data can be accessed from GEO repository under an accession number GSE158298 [29]), summary statistics of the COVID-19 GWAS (round 5) by the COVID-19 Host Genetics Initiative are available at https://www.covid19hg.org/results/r5/ [30], eQTL summary statistics can be downloaded from the eQTL Catalogue [31] and the GTEx Portal [32], and the variant calls from 1000 Genomes Project on the GRCh38 reference assembly from the EBI FTP site [33]. Analysis code has been deposited to the GitHub repository at https://github.com/LappalainenLab/covid-crispr-eqtl [34] under an Apache 2.0 License and at the repository Zenodo [35].
