# Supplementary material for: Integrative approach identifies SLC6A20 and CXCR6 as putative causal genes for the COVID-19 GWAS signal in the 3p21.31 locus
Source: Genome Biol. 2021 Aug 23;22:242. doi: 10.1186/s13059-021-02454-4 (PMC8381345; doi:10.1186/s13059-021-02454-4)
Supplement: Supplementary file 1 — Additional file 1:. Supplementary figures S1-S5. [file 13059_2021_2454_MOESM1_ESM.pdf]

Supplementary Figures for

Integrative approach identifies *SLC6A20* and *CXCR6* as putative causal genes for the COVID-19 GWAS signal in the 3p21.31 locus

Silva Kasela[1,2,*], Zharko Daniloski[1,3], Sailalitha Bollepalli[1], Tristan X. Jordan[4], Benjamin R. tenOever[4], Neville E. Sanjana[1,3], Tuuli Lappalainen[1,2,*]

1 - New York Genome Center, New York, NY, USA
2 - Department of Systems Biology, Columbia University, New York, NY, USA
3 - Department of Biology, New York University, New York, NY, USA
4 - Department of Microbiology, Icahn School of Medicine at Mount Sinai, New York, NY, USA

*Correspondence to: skasela@nygenome.org (S.K.), tlappalainen@nygenome.org (T.L.)

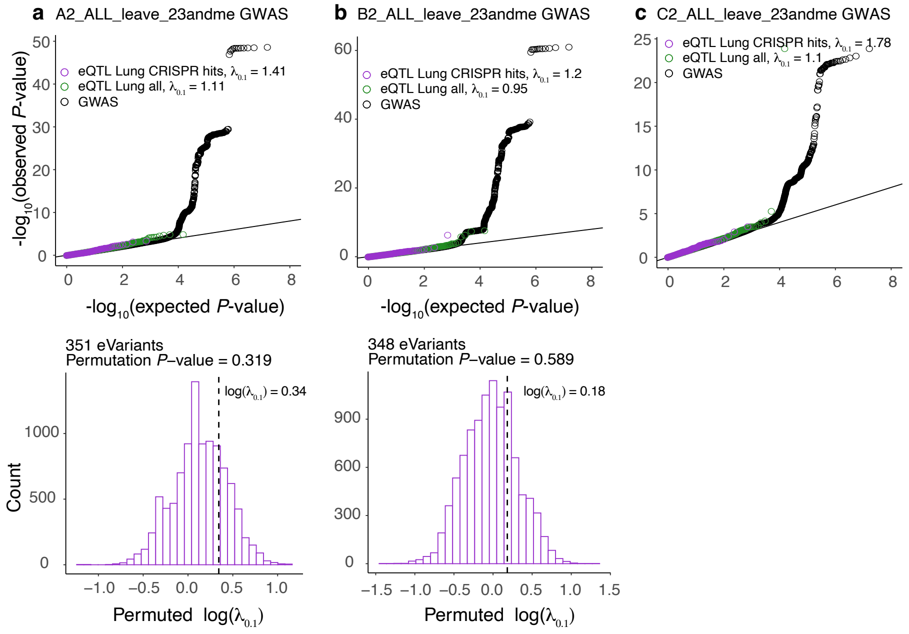

Figure S1. Inflation of COVID-19 GWAS signal for GTEx Lung eQTLs for the top-ranked genes from the CRISPR screen. The top panels shows the Q-Q plots comparing the expected and observed *P*-value distribution of COVID-19 GWAS *P*-value ($-\log_{10}$) for all the variants tested in GWAS (black), variants that are lead eQTLs in GTEx Lung (green), variants that are lead eQTLs in GTEx Lung for the top-ranked genes from the CRISPR screen (purple) for the following COVID-19 phenotypes (COVID-19 worldwide meta-analysis without 23andMe by COVID-19 Host Genetics Initiative, freeze 5 data): (**a**) critical illness (A2), hospitalization (B2), and reported infection (C2) as compared to population controls. Inflation estimate $\lambda_{0.1}$ measures the inflation of test statistics relative to the chi-square quantile function of 0.1. *i.e.*, 10% of the most significant tests. The bottom panel shows the histogram of the permuted $\log(\lambda_{0.1})$ estimate to test the significance of the inflation of variants that are *cis*-eQTLs for the top-ranked genes from the CRISPR screen in GTEx Lung in the given COVID-19 GWAS together with the two-sided permutation *P*-value. Vertical dashed line denotes the observed $\log(\lambda_{0.1})$ value. Histogram for reported infection is shown on Fig. 1a.

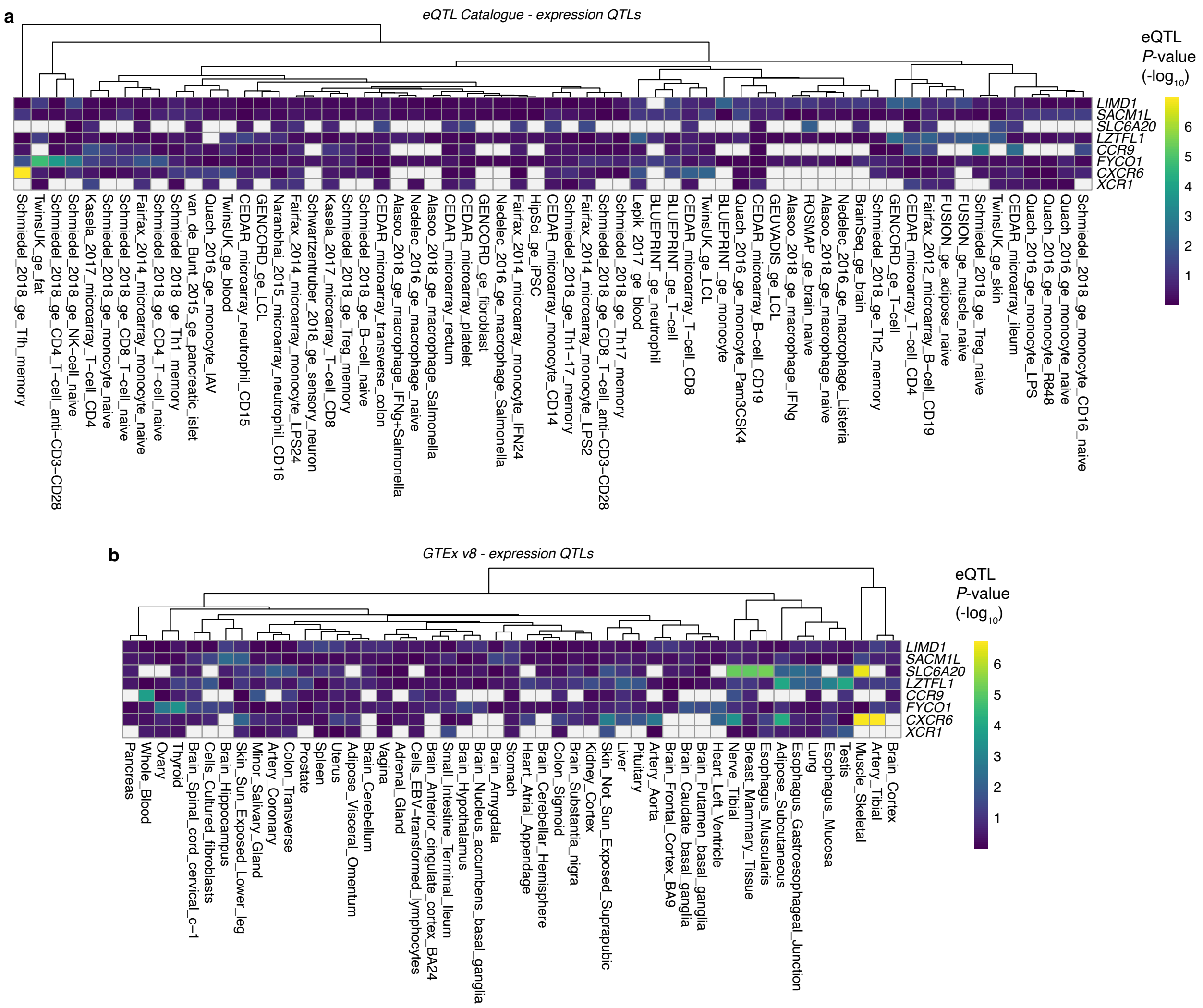

Figure S2. **The lead GWAS variant as an *cis*-eQTL for the eight genes in the 3p21.31 locus associated with COVID-19 GWAS**. Heatmaps showing the *cis*-eQTL *P*-value of the lead GWAS variant rs10490770 from the hospitalized COVID-19 vs population GWAS (B2 worldwide meta-analysis with 23andMe) for the eight genes in the 3p21.31 locus in 63 cell types and tissues from the eQTL Catalogue (**a**) and 49 tissues from GTEx v8 (**b**). A tile is colored in white, if there was no data for the variant-gene pair in the given eQTL data set (*e.g.*, the gene was not tested in *cis*-eQTL mapping). eQTL data sets are clustered using the Euclidean distance and complete linkage method.

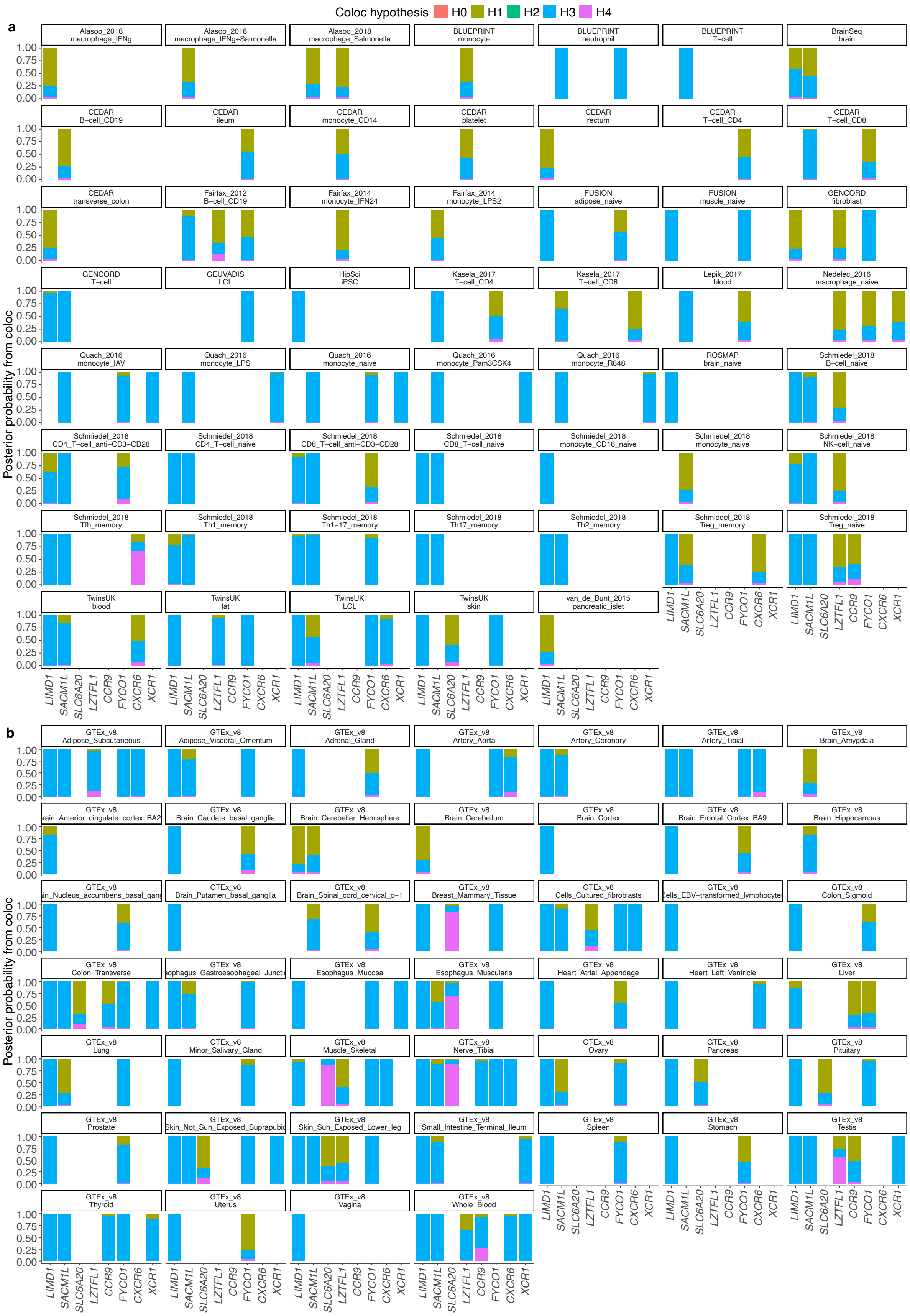

Figure S3. **Summary of colocalization analysis of hospitalized COVID-19 GWAS and *cis*-eQTLs in**

different cell types and tissues with coloc. Posterior probability (*y*-axis) for the five different hypothesis H0 (no association), H1 (association in GWAS), H2 (association in eQTL), H3 (association both in GWAS and eQTL, two independent SNPs), H4 (association both in GWAS and eQTL, one shared SNP) from coloc is shown for each of the eight genes (*x*-axis) used in the colocalization analysis with *cis*-eQTLs from the eQTL Catalogue (**a**) and GTEx v8 (**b**). For eQTL studies using microarray technology with multiple probes mapping to the same gene, we chose the probe which resulted in the lowest *cis*-eQTL *P*-value for the lead COVID-19 GWAS variant rs10490770 (B2 worldwide meta-analysis with 23andMe) to represent the gene. Also, there is a suggestive signal for colocalization with *cis*-eQTLs for *LZTFL1* in Testis in GTEx, but the *cis*-eQTL signal is not statistically significant (FDR > 0.05) and the lead *cis*-eQTL variant has GWAS *P*-value = 0.1975 in the B2 GWAS without 23andme.

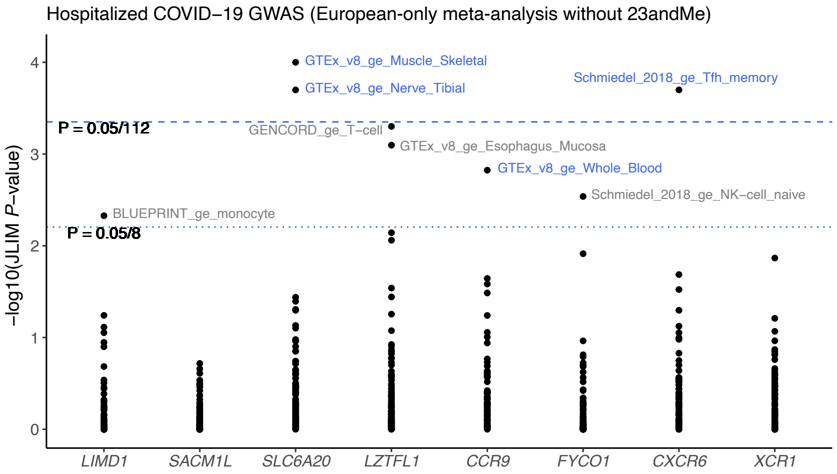

Figure S4. **Summary of colocalization analysis of hospitalized COVID-19 GWAS and *cis*-eQTLs in different cell types and tissues with JLIM**. Estimated joint likelihood *P*-values (*y*-axis) are shown for the eight genes (*x*-axis) analyzed for the hospitalized COVID-19 GWAS (B2 European-only meta-analysis without 23andMe) signal in the 3p21.31 locus. Highlighted are tissues with a significant *P*-value for colocalization with a lenient significance threshold accounting only for the number of genes tested ($P < 0.05/8$). Tissues with minimum *cis*-eQTL *P*-value $>10^{-4}$ for a given gene are colored in grey.

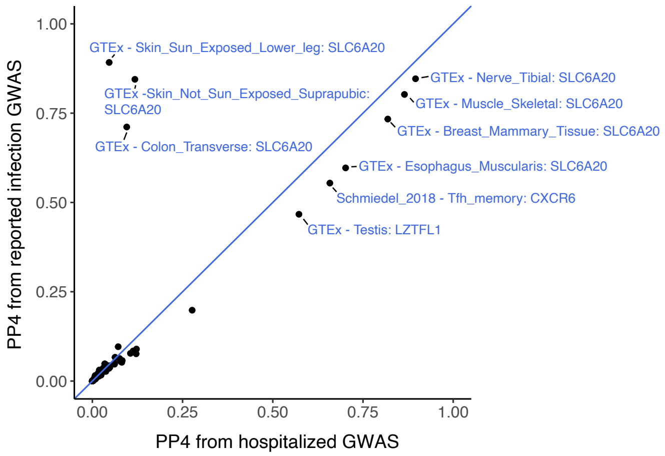

Figure S5. **Comparison of colocalization results between COVID-19 hospitalization and COVID-19 susceptibility GWAS with *cis*-eQTLs using coloc.** Posterior probability for shared causal variant in the eQTL and GWAS study (PP4) are shown for hospitalized COVID-19 GWAS (worldwide meta-analysis without 23andMe, *x*-axis) and reported SARS-CoV-2 infection GWAS (worldwide meta-analysis without 23andMe, *y*-axis). Note that there are several independent signals present in the reported infection GWAS that remain unresolved such that the COVID-19 Host Genetics Initiative cautions against interpretation of this complex signal with present data [1]. Also, the coloc results should be interpreted with caution as the one causal variant assumption is violated.

References

1. The COVID-19 Host Genetics Initiative, Ganna A. Mapping the human genetic architecture of COVID-19 by worldwide meta-analysis. Preprint at medRxiv https://doi.org/10.1101/2021.03.10.21252820. 2021.
