# Supplementary material for: Integrative approach identifies SLC6A20 and CXCR6 as putative causal genes for the COVID-19 GWAS signal in the 3p21.31 locus
Source: Genome Biol. 2021 Aug 23;22:242. doi: 10.1186/s13059-021-02454-4 (PMC8381345; doi:10.1186/s13059-021-02454-4)
Supplement: Supplementary file 3 — Additional file 3. Peer review history. [file 13059_2021_2454_MOESM3_ESM.docx]

Review History

**First round of review**

**Reviewer 1**

**Are you able to assess all statistics in the manuscript, including the appropriateness of statistical tests used?**

Yes, and I have assessed the statistics in my report.

**Comments to author:**

In the manuscript entitled "Integrative approach identifies SLC6A20 and CXCR6 as putative causal genes for the COVID-19 GWAS signal in the 3p21.31 locus", Kasela et al made use of public eQTL datasets and a CRISPR screen to find putative gene mediators for COVID-19 genetic susceptibility. I found the authors used creatively these datasets, the manuscript is well written, and the methods are well described and clear. Additionally, the authors are addressing an important question.

While I appreciate that the authors are in general careful with their language/claims throughout the manuscript, I think that the sentence "Nevertheless, our results *establish* the link between genes enriched in the in vitro CRISPR screen and the genetic component of COVID-19 risk in humans." might be an overstatement. The authors found a marginally significant enrichment in hospitalization genetic association for the lung eQTL genes enriched in the CRISPR screen. However, this signal was not replicated in the other GWAS cohorts. The authors argue this could be due to samples size, although the GWAS enrichment of low P-values actually looks stronger for several of the other cohorts (black dots). And the authors do their co-localization analysis in a different GWAS cohort that didn't have the lung CRISPR-eQTL enrichment signal. Can the authors list the samples sizes for each GWAS cohort? Could the authors perform complementary analyses that confirm the signal found in the hospitalization GWAS is robust? For example, the authors could use the LD score regression method by Finucane et al (Nat Genet 2015) to perform heritability enrichment analysis at the significant CRISPR screen genes (+/- 100kb or a similarly sized window).

As a side comment, I wonder if the authors observe the signal only in the hospitalization GWAS because for that particular phenotype (hospitalization vs not, given you already got infected) the genetic susceptibility might be more lung-driven, whereas the phenotypes captured by the other GWAS cohorts might be driven by other tissue/cell-types?

In the eQTL-GWAS co-localizaton analyses the authors found a suggestive signal with coloc. Could the authors use additional co-localization algorithms to see if this is supported by other methods? For example, the JLIM method by Chun et al (Nat Genet 2017) is supposed to be superior to coloc.

Could the authors comment on whether the GWAS risk allele is associated with higher or lower expression of CXCR6? And likewise for SLC6A20?

It is a bit unexpected that the co-localized eQTLs are not for lung given the CRISPR screen was made in lung cells. Did the authors look for co-localization with splicing QTLs?

Could the authors also comment on why they think the CXCR6 CRISPR ranking was better for low dosages of the virus compared to high dosages?

**Reviewer 2**

**Are you able to assess all statistics in the manuscript, including the appropriateness of statistical tests used?**

Yes, and I have assessed the statistics in my report.

**Comments to author:**

In this paper, the authors present an integrative analysis of a locus on chromosome 3 which has been associated with COVID-19 susceptibility and severity in prior GWAS work. They combine results from a CRISPR screen aimed at determining genes that are necessary for infection with COVID-19, GWAS data from several studies looking at COVID-19 susceptibility and severity, and publicly available eQTL data sets. They first demonstrate that eQTLs for genes identified in the CRISPR screen show enriched association signal in one of the six prior published GWAS. They then go on to take the peak variant in a region on chromosome 3 from another GWAS and use the CRISPR assay results as well as eQTL data to determine the candidate functional genes under this GWAS peak. Overall, I think the study is well-executed and a valuable addition to the literature illustrating how integrative analyses can help illuminate functional genes under a GWAS peak. However, there are a few places where a lack of precise language makes following the thread of the paper unnecessarily difficult, which I hope the authors can address.

Major revisions:

1. While I appreciate the analysis presented and think it is a useful addition to the paper, the first paragraph of the Results section seems to come out of nowhere after all the focus on the Chr 3 locus in the abstract and intro. And the vagueness of the first sentence does not help alleviate the intitial confusion upon reading it. I'm not sure how your analysis assesses how top-ranked genes from the CRISPR screen "mediate disease risk in humans". You are not performing any kind of mediation analysis here. I would suggest revising this sentence to be something like: "To set the stage for our analysis of the chromosome 3p21.31 locus, we first wanted to assess whether genetic regulatory variants for genes pinpointed.... (continuing with second sentence as it is now)"

For this analysis, how exactly did you select the 588 genes from the CRISPR screen? What does it mean to be "top ranked"? (Since 588 is not a nice even number or anything, I presume there was some specific criteria for selecting this set.) This selection step is not clear from the methods, even referring to the attached preprint.

2. The main analysis of the paper would benefit from some further context and motivation. Why was this chromosome 3p21.31 locus selected? You state that "several studies have identified a rubust association" at this locus, but that is not the same as it being genome-wide significant or the most significant locus from these studies. This is maybe a just a lack of precision, and it is also OK if this region was selected because it had some compelling candidate genes, regardless of significance from the GWAS. But the motivation should just be described more clearly.

Next, why was the selected SNP the lead SNP in this region from the ANA_C2_V2 study specifically? Reading the Methods, I can gather it was because this was the largest study; please clarify this briefly in the Results as well. Since this is not the study that showed enrichment of GWAS signal at lung eQTLs targeting high-ranked CRISPR genes from the previous paragraph, it is somewhat confusing as to why ANA_C2_V2 is used to select this peak variant on first reading this paragraph. Since this variant is the key determinant of the rest of the analyses presented in the paper, additional motivation for selecting it would be good.

3. From the Discussion, I'm not sure why the fact that there were eQTLs in different tissues for SLC6A20 and CXCR6 indicates "extrapulmonary spread of SARS-CoV-2": perhaps these genes just play a role in these tissues that has nothing to do with SARS-CoV-2?

4. From the Conclusion: The first sentence is a bit of a stretch. You are not showing that these genes contribute to susceptibility, but that they are reasonable candidates based on your integrative analysis. So something like "We demonstrated that genes required for SARS-CoV-2 infection in vitro are a useful filter for refining genetic association results related to COVID-19 susceptibility in humans." would be reasonable to say, in my opinion.

5. From the Methods, "Prioritization of genes in the 3p21.31 region..." you state that you prioritized genes "based on" p-value in eqtl studies (p5 line 28). What does this mean exactly? You picked the genes with the lowest eQTL p-values? Please be more precise here.

Minor changes:

For Fig 1e, the tissue should be given for each gene as part of the panel titles

p1 line 56: mean -> means

p1 line 58: trait -> traits

p2 line 23: study -> studies

p4 line 60: should be "274 out of 588 top-"

**Authors Response**

**Point-by-point responses to the reviewers’ comments:**

*We thank both the reviewers for their feedback. We have responded to each of the reviewer points below and updated the manuscript accordingly.*

*Please note that COVID-19 Host Genetics Initiative (HGI) has released two additional data freezes after the submission of this manuscript and no longer provides summary statistics for some of the COVID-19-related phenotypes. We have updated our analysis to use the latest data freeze (release 5 from January 2021) to show the robustness of our results, and analyze only the three main COVID-19 phenotypes for simplicity to the reader (critically ill, hospitalized, reported infection as compared to population controls) in this revised manuscript.*

*Also, in light of the newly found additional independent signals in the 3p21.31 locus for COVID-19 susceptibility and the complex nature of this locus, we have shifted our main focus on the hospitalization due to COVID-19 GWAS. We present the colocalization results for COVID-19 susceptibility GWAS in the supplement and do not interpret this signal further as the present data is not sufficient to resolve the structure of the locus as cautioned by the COVID-19 HGI (*[*https://www.medrxiv.org/content/10.1101/2021.03.10.21252820v2*](https://www.medrxiv.org/content/10.1101/2021.03.10.21252820v2)*).*

Reviewer #1:

In the manuscript entitled "Integrative approach identifies SLC6A20 and CXCR6 as putative causal genes for the COVID-19 GWAS signal in the 3p21.31 locus", Kasela et al made use of public eQTL datasets and a CRISPR screen to find putative gene mediators for COVID-19 genetic susceptibility. I found the authors used creatively these datasets, the manuscript is well written, and the methods are well described and clear. Additionally, the authors are addressing an important question.

Q1: While I appreciate that the authors are in general careful with their language/claims throughout the manuscript, I think that the sentence "Nevertheless, our results *establish* the link between genes enriched in the in vitro CRISPR screen and the genetic component of COVID-19 risk in humans." might be an overstatement. The authors found a marginally significant enrichment in hospitalization genetic association for the lung eQTL genes enriched in the CRISPR screen. However, this signal was not replicated in the other GWAS cohorts. The authors argue this could be due to samples size, although the GWAS enrichment of low P-values actually looks stronger for several of the other cohorts (black dots). And the authors do their co-localization analysis in a different GWAS cohort that didn't have the lung CRISPR-eQTL enrichment signal. Can the authors list the samples sizes for each GWAS cohort? Could the authors perform complementary analyses that confirm the signal found in the hospitalization GWAS is robust? For example, the authors could use the LD score regression method by Finucane et al (Nat Genet 2015) to perform heritability enrichment analysis at the significant CRISPR screen genes (+/- 100kb or a similarly sized window).

*Answer: Thank you for these comments and suggestions! We have performed additional analyses to explore the enrichment of top-ranked genes from the CRISPR screen in the genetic component of COVID-19 risk in humans. We used stratified LD score regression to estimate the heritability enrichments for the three main COVID-19 phenotypes (critically ill, hospitalized, reported infection as compared to population controls) in the top-ranked genes conditional on the 96 annotations in the baseline model and all protein-coding genes. We again observe a suggestive signal for one of the COVID-19 phenotypes - enrichment P = 0.068 for hospitalized COVID-19 (P = 0.104 for critical illness and P = 0.485 for reported infection).*

Please note that the list of top-ranked genes from the CRISPR screen used in the revised manuscript is slightly different (please see the answer to Reviewer’s 2 first question). In freeze 5 data with the newer set of top-ranked genes, we observe a suggestive support for inflation of SARS-CoV-2 reported infection GWAS signal in the lung (two-sided permutation P-value = 0.0698). We have updated the manuscript with the SNP heritability results based on partitioned LDSC, and we have moved the QQ-plot presented on Fig. 1 to the supplementary to highlight the partitioned LDSC results for the reported infection on Fig 1.

*We agree with the reviewer that while these results support the link between genes enriched in the in vitro CRISPR screen and the genetic component of COVID-19 risk in humans, future COVID-19 GWASes with larger sample sizes would be needed to establish the link. We have edited our conclusions based on the genome-wide analysis in the Introduction and Conclusion sections to highlight that we observe a suggestive association between the top-ranked CRISPR genes contributing to COVID-19 phenotypes.*

*Also, sample sizes for the COVID-19 GWAS cohorts are listed in the Methods section in the new section entitled “COVID-19 phenotypes and GWAS“.*

Q2: As a side comment, I wonder if the authors observe the signal only in the hospitalization GWAS because for that particular phenotype (hospitalization vs not, given you already got infected) the genetic susceptibility might be more lung-driven, whereas the phenotypes captured by the other GWAS cohorts might be driven by other tissue/cell-types?

*Answer: We thank the reviewer for this interesting question. Unfortunately, the present data do not seem to be sufficient to answer this question. In the revised manuscript we have focused on the three main COVID-19 phenotypes only (see the note at the beginning of the response letter), but we run the LDSC analysis for B1 phenotype (hospitalized covid vs not hospitalized covid) to prove an answer to this question. We observe very large standard errors for B1 (enrichment = -3.31, se = 1867.92), probably highlighting lower power for this phenotype compared to the others that use population controls.*

*We include inflation testing results for B1 here for the reviewer to show that the suggestive signal is present in Freeze 5 data: perm P = 0.034 - Freeze 3, old set of top-ranked genes; perm P = 0.166 - Freeze 5, old set of top-ranked genes, worldwide meta-analysis; perm P = 0.069 - Freeze 5, new set of top-ranked genes, European-only meta-analysis. However, the inflation signal is weaker and not nominally significant based on one-sided permutation P-values. Hopefully, next freezes of COVID-19 GWAS would be helpful to understand the reasons behind this.*

Q3: In the eQTL-GWAS co-localizaton analyses the authors found a suggestive signal with coloc. Could the authors use additional co-localization algorithms to see if this is supported by other methods? For example, the JLIM method by Chun et al (Nat Genet 2017) is supposed to be superior to coloc.

*Answer: Thank you for this suggestion! We have now performed colocalization analysis using the JLIM method with default options to confirm the observed colocalization signals for CXCR6 and SLC6A20, and observed similar results based on the two analyses. There were additional colocalization signals with a lenient P-value threshold for multiple testing P < 0.05/8 (correcting for the tested genes only), but in all 5 cases the minimum eQTL P-value in the region or the lead GWAS variant had eQTL P-value > 1e-4 (note that we had excluded genes with minimum eQTL P-value > 1e-4 from coloc analysis). We have updated the manuscript to include these results, and show the results in Additional file 1: Figure S4 and Additional file 2: Table S2.*

Q4: Could the authors comment on whether the GWAS risk allele is associated with higher or lower expression of CXCR6? And likewise for SLC6A20?

*Answer: The alternative allele of the GWAS variant that is increasing the risk for hospitalization due to COVID-19 is associated with lower expression of CXCR6 and higher expression of SLC6A20 in the respective tissues, where we observe colocalization. We have clarified this in the text and added the beta estimates and standard errors of the lead GWAS variant and lead eQTL to Additional file 2: Table S1 as well.*

Q5: It is a bit unexpected that the co-localized eQTLs are not for lung given the CRISPR screen was made in lung cells. Did the authors look for co-localization with splicing QTLs?

*Answer: We have now included splicing QTLs (sQTLs) from GTEx v8 and transcript usage (tuQTLs) from the eQTL Catalogue to the colocalization analysis with coloc. We do not observe evidence for colocalization with any of these molQTLs and the hospitalized COVID-19 GWAS signal. We have updated the revised manuscript to include these results and share the results as Additional file 2: Table S3.*

Q6: Could the authors also comment on why they think the CXCR6 CRISPR ranking was better for low dosages of the virus compared to high dosages?

*Answer: We thank the reviewer for this question. It is an interesting observation that CXCR6 ranking is better for low versus high viral doses. Previous studies have shown that there is a positive correlation between the viral dose and disease severity in patients infected with coronaviruses and influenza (reviewed by Damme et al., 2021, doi: 10.1016/j.mehy.2020.110431), suggesting that variations in viral doses can elicit different cellular responses. The CRISPR screen identified that CXCR6 chemokine receptor is required for SARS-CoV-2 infection and its deficiency provides a protective function against SARS-CoV-2 at low viral doses. The protective role was not as prominent at high viral doses, likely due to additional cellular mechanisms that may be in place under higher viral pressure.*

*However, CXCR6 and SLC6A20 could have a role in different COVID-19 phenotypes in light of the other independent variants found for the reported infection GWAS as noted at the beginning of the response letter. To be up-to-date, we have included this new observation to the revised manuscript.*

*New edits to Results and Discussion:*

*“Notably, in addition to the well replicated signal for COVID-19 severity in the 3p21.31 locus, other independent signals more strongly associated with susceptibility to SARS-CoV-2 infection in the same region have been identified as sample sizes for COVID-19 GWAS have grown [20]. The two candidate genes based on our integrative analysis, SLC6A20 and CXCR6, have been prioritized for reported infection and more severe outcomes of SARS-CoV-2 infection, respectively [5]. Corroborating evidence from entirely independent approaches pinpointing SLC6A20 and CXCR6 presented here, together with additional evidence from other studies, warrants further research into exploring the virological or mechanistic mode of action of these two genes in understanding their role in COVID-19 susceptibility and severity.”*

Reviewer #2:

In this paper, the authors present an integrative analysis of a locus on chromosome 3 which has been associated with COVID-19 susceptibility and severity in prior GWAS work. They combine results from a CRISPR screen aimed at determining genes that are necessary for infection with COVID-19, GWAS data from several studies looking at COVID-19 susceptibility and severity, and publicly available eQTL data sets. They first demonstrate that eQTLs for genes identified in the CRISPR screen show enriched association signal in one of the six prior published GWAS. They then go on to take the peak variant in a region on chromosome 3 from another GWAS and use the CRISPR assay results as well as eQTL data to determine the candidate functional genes under this GWAS peak. Overall, I think the study is well-executed and a valuable addition to the literature illustrating how integrative analyses can help illuminate functional genes under a GWAS peak. However, there are a few places where a lack of precise language makes following the thread of the paper unnecessarily difficult, which I hope the authors can address.

Major revisions:

1. While I appreciate the analysis presented and think it is a useful addition to the paper, the first paragraph of the Results section seems to come out of nowhere after all the focus on the Chr 3 locus in the abstract and intro. And the vagueness of the first sentence does not help alleviate the intitial confusion upon reading it. I'm not sure how your analysis assesses how top-ranked genes from the CRISPR screen "mediate disease risk in humans". You are not performing any kind of mediation analysis here. I would suggest revising this sentence to be something like: "To set the stage for our analysis of the chromosome 3p21.31 locus, we first wanted to assess whether genetic regulatory variants for genes pinpointed.... (continuing with second sentence as it is now)"

For this analysis, how exactly did you select the 588 genes from the CRISPR screen? What does it mean to be "top ranked"? (Since 588 is not a nice even number or anything, I presume there was some specific criteria for selecting this set.) This selection step is not clear from the methods, even referring to the attached preprint.

*Answer: Thanks for these comments and suggestions! We have revised the text to include more focus on the CRISPR screen in the Introduction and rephrased the first sentence of the Results section.*

*New additions to the Introduction: “Top-ranked genes from this CRISPR screen have an established link in SARS-CoV-2 infection in a cell line, and could be informative for prioritizing host genes for COVID-19.”*

*New additions to the Results and Discussion: “To set the stage for identifying the causal genes for COVID-19 in the 3p21.31 locus, we first evaluated whether the top-ranked genes from the CRISPR screen in human lung cells contribute to COVID-19 risk in humans.”*

*We apologize for the lack of precision regarding the top-ranked genes. The 588 genes used for the GWAS inflation analysis were from the initial list of top-ranked genes that were enriched in at least one screen (low or high MOI setting). In the final version of the Daniloski et al. manuscript (https://doi.org/10.1016/j.cell.2020.10.030), this particular list of genes is not highlighted. To be concordant with the data presented in Daniloski et al., we formed our list of top-ranked genes for the inflation and partitioned heritability analysis based on the gene list presented on Figure S1C “Overlap of top-ranked (top 500) genes between 3 different analysis methods (RRA, RIGER, and SBR). 142 genes are found by all 3 methods.” For example, 355 out of 588 (60.4%) genes from the initial list are part of the newer list formed based on low MOI experiment. We have updated the “Genome-wide CRISPR screen and guide RNA analysis” section to include more details about the ranking of the genes and the list of top-ranked genes used for the enrichment analysis.*

2. The main analysis of the paper would benefit from some further context and motivation. Why was this chromosome 3p21.31 locus selected? You state that "several studies have identified a rubust association" at this locus, but that is not the same as it being genome-wide significant or the most significant locus from these studies. This is maybe a just a lack of precision, and it is also OK if this region was selected because it had some compelling candidate genes, regardless of significance from the GWAS. But the motivation should just be described more clearly.

Next, why was the selected SNP the lead SNP in this region from the ANA_C2_V2 study specifically? Reading the Methods, I can gather it was because this was the largest study; please clarify this briefly in the Results as well. Since this is not the study that showed enrichment of GWAS signal at lung eQTLs targeting high-ranked CRISPR genes from the previous paragraph, it is somewhat confusing as to why ANA_C2_V2 is used to select this peak variant on first reading this paragraph. Since this variant is the key determinant of the rest of the analyses presented in the paper, additional motivation for selecting it would be good.

*Answer: We have revised the text to be more precise in motivating the use of the chromosome 3p21.31 locus as the main interest of our manuscript.*

*Revised text: “Most notably, the chromosome 3p21.31 locus was one of the first genome-wide significant loci found to be associated with COVID-19 [3]. To date, 3p21.31 encompasses the strongest and the most replicated signal for more severe outcomes of SARS-CoV-2 infection.”*

*As we note at the beginning of the response letter, we have shifted our focus on the hospitalized COVID-19 GWAS, but we would like to give some additional reasons to the reviewer: C2 or the reported infection GWAS was initially chosen because it had the largest sample size in Freeze 3 data. However, as the COVID-19 GWASes have become more powerful due larger sample sizes since October 2020, when we first submitted the manuscript, it has been shown now that the association signal in the 3p21.31 locus that was first reported by Ellinghaus et al. 2020 in June is linked with more severe outcomes of SARS-CoV-2 infection, such as hospitalization or respiratory failure. Thus, the hospitalized COVID-19 GWAS is a better match compared to reported infection GWAS.*

*We have clarified the connection between 3p21.31 and COVID-19 severity in the Introduction as noted in response to the previous question to motivate using hospitalization COVID-19 GWAS for performing colocalization analysis. For transparency we show the colocalization results for COVID-19 susceptibility in the supplement (Additional file 1: Figure S5), but do not interpret this further as the present data is not sufficient as cautioned by the COVID-19 Host Genetics Initiative (https://www.medrxiv.org/content/10.1101/2021.03.10.21252820v2) - most importantly the colocalization events observed in Freeze 3 data are observed in the latest Freeze 5 data for both of the COVID-19 phenotypes.*

*Also, we have included a separate paragraph for the COVID-19 GWAS in the Methods section (“COVID-19 phenotypes and GWAS”) to clarify the selection of the lead GWAS variant highlighted on Fig. 1c-e.*

3. From the Discussion, I'm not sure why the fact that there were eQTLs in different tissues for SLC6A20 and CXCR6 indicates "extrapulmonary spread of SARS-CoV-2": perhaps these genes just play a role in these tissues that has nothing to do with SARS-CoV-2?

*Answer: We agree that further research is needed to establish whether all these eQTs have a causal role in COVID-19 risk. We have added a sentence as a clarification to the revised manuscript: ”However, further research is needed to establish whether all these eQTLs have a causal role in COVID-19 risk.”*

4. From the Conclusion: The first sentence is a bit of a stretch. You are not showing that these genes contribute to susceptibility, but that they are reasonable candidates based on your integrative analysis. So something like "We demonstrated that genes required for SARS-CoV-2 infection in vitro are a useful filter for refining genetic association results related to COVID-19 susceptibility in humans." would be reasonable to say, in my opinion.

*Answer: The original sentence: “In this work, we demonstrated that genes required for SARS-CoV-2 infection in vitro also contribute to COVID-19 susceptibility in humans.” actually referred to the genome-wide enrichment of COVID-19 risk among the top-ranked genes from the CRISPR screen, but this was indeed unclear. We have revised the sentence to: “In this work, our genome-wide analysis suggested that genes required for SARS-CoV-2 infection in vitro also contribute to COVID-19 susceptibility in humans.”*

5. From the Methods, "Prioritization of genes in the 3p21.31 region..." you state that you prioritized genes "based on" p-value in eqtl studies (p5 line 28). What does this mean exactly? You picked the genes with the lowest eQTL p-values? Please be more precise here.

*Answer: We have revised the text to clarify that prioritization of genes was done by ranking of the genes according to the p-value observed in the eQTL studies.*

Minor changes:

For Fig 1e, the tissue should be given for each gene as part of the panel titles

p1 line 56: mean -> means

p1 line 58: trait -> traits

p2 line 23: study -> studies

p4 line 60: should be "274 out of 588 top-"

*Answer: Thanks for the suggestion for Fig. 1e and pointing out these typos! We have updated the panel titles and fixed typos.*

**Second round of review**

**Reviewer 1**

The authors have satisfactorily addressed my comments. Great work!

**Reviewer 2**

The authors have done a nice job responding to the comments from the reviewers and the paper is improved in terms of clarity and robustness of results presented. I noticed one small inconsistency that I wanted to point out.

Minor revisions:

p5 line 49: should that be with or without 23AndMe? I believe everywhere else in the paper, you are excluding 23AndMe from the analysis (although I'm not completely sure why) and I found this detail confusing.
